# * In vitro* evaluation of antifungal activity of monolaurin against *Candida albicans* biofilms

**DOI:** 10.7717/peerj.2148

**Published:** 2016-06-22

**Authors:** Dalia Seleem, Emily Chen, Bruna Benso, Vanessa Pardi, Ramiro M. Murata

**Affiliations:** 1 Herman Ostrow School of Dentistry, Division of Periodontology Diagnostic Sciences, Dental Hygiene and Biomedical Science, University of Southern California, Los Angeles, CA, United States; 2 Piracicaba Dental School, University of Campinas, Piracicaba, Sao Paulo, Brazil; 3 Current affiliation: School of Dentistry, Faculty of Medicine, Universidad Austral de Chile, Valdivia, Chile

**Keywords:** *Candida albicans*, *In vitro*, Antimicrobial agent, Monolaurin, Biofilms, Virulence factors, Proteolytic enzymes, Oral candidiasis, MIC/MFC, Host inflammatory response

## Abstract

Monolaurin (also known as glycerol monolaurate) is a natural compound found in coconut oil and is known for its protective biological activities as an antimicrobial agent. The nature of oral candidiasis and the increased antifungal resistance demand the search for novel antifungal therapeutic agents. In this study, we examine the antifungal activity of monolaurin against *Candida albicans* biofilms (strain ATCC:SC5314/MYA2876) *in vitro* and investigate whether monolaurin can alter gene expression of host inflammatory cytokines, IL-1*α* and IL-1*β*. In a co-culture model, oral fibroblast cells were cultured simultaneously with *C. albicans* for 24 hrs followed by the exposure to treatments of monolaurin (3.9–2,500 µM), positive control fluconazole (32.2 µM), and vehicle control group (1% ethanol), which was a model used to evaluate the cytotoxicity of monolaurin on fibroblasts as well as to analyze morphological characteristics of biofilms through fluorescence microscopy. In addition, the co-culture model was used for RNA extraction of oral fibroblasts to assess gene expression of host inflammatory cytokines, using quantitative real-time PCR. Our results showed the MIC and MFC of monolaurin were in the range 62.5–125 µM and 125–250 µM, respectively. Biofilm antifungal assay showed significant reduction in Log (CFU/ml) of biofilms treated with 1,250 and 2,500 µM of 1-monolaurin when compared to the control groups . There was also a significant down-regulation of IL-1*α* and IL-1*β* in the co-culture treated with monolaurin. It can be concluded that monolaurin has a potential antifungal activity against *C. albicans* and can modulate the pro-inflammatory response of the host.

## Introduction

*Candida albicans* is a prevalent opportunistic human fungal pathogen that lives commensally in the gut, oral pharyngeal, genito-urinary tract and skin. However, it may become pathogenic in immunocompromised patients or in individuals with an imbalance of competing bacterial microflora (Berman, 2012; Dovigo et al., 2011b; Neppelenbroek et al., 2006). The disseminated forms of the disease can be life-threatening with mortality rates of 40–60% among immunocompromised, cancer patients, or individuals exposed to multiple treatments, such as broad spectrum antibiotics, chemotherapy, immunosuppressive therapy, and anti-retroviral therapy (Dovigo et al., 2011a; Krcmery & Barnes, 2002; Neppelenbroek et al., 2006). The pathogenicity of the *Candida* species is attributed to critical virulence factors, such as adherence to surfaces (on both tissues and medical devices), biofilm formation, evasion of host immune defense mechanisms, and secretion of proteolytic enzymes, such as secreted aspartyl proteases (*SAP*) and phospholipases (Correia et al., 2010).

As mentioned earlier, there are a number of virulence factors associated with the pathogenicity of *Candida albicans*, such as biofilm formation and secretion of proteolytic enzymes, e.g., secreted aspartic proteases (*SAPs*), phospholipase, and lipase enzymes. Mature fungal biofilms are characterized by a dense community of both yeasts and hyphae structures encased in a thick extracellular polymeric substance (EPS), which ensures adequate diet is supplied to biofilms, transports waste products, and may also have a role in the antifungal resistance of *Candida* species (Ramage et al., 2001). The gene expression of *SAP*s (1–10) family as a whole has been associated with tissue damage and host invasion of the pathogenic *C. albicans* (Lermann & Morschhauser, 2008; Nailis et al., 2010). Hyphal formation is considered the most critical factor involved in epithelial invasion and the degradation of epithelial cell junction proteins (Naglik et al., 2008; Villar et al., 2007).

Immune host defense plays a critical role in the phagocytosis of the pathogenic *Candida* species during fungal infections. Activation of the CD4+ T cells results in the production of pro-inflammatory chemokines and cytokines, such as IL-1*α*, IL-1*β*, and TNF-*α*. Antigen uptake through activation of anti-inflammatory cytokines IL-10 via dendritic cells (DCs) during the later phase of infection, ultimately leads to the elimination of the fungus (Chandra et al., 2007; Gresnigt et al., 2012). Thus, the pathogenic state of candidiasis is marked by an increase in the pro-inflammatory response, followed by an increase in the anti-inflammatory cytokines.

Current treatments for *C. albicans* infection consist of topical and systemic pharmaceutical antifungal agents, with triazoles being the first line of defense effective against most *Candida* species (Samaranayake & Macfarlane, 1990). However, due to the limited number of antifungal treatments available and the widespread use of such agents, there has been an increase in antifungal resistance (Hunter et al., 1998; White, Marr & Bowden, 1998). For instance, oropharyngeal candidiasis in patients with advanced HIV infection and AIDS is usually treated with fluconazole. However, it is often difficult to completely eradicate the infection without relapse occurring within a few months (Rex, Rinaldi & Pfaller, 1995). Therefore, the increase in both fungal infections and antifungal resistance demand the search for new and effective antifungal therapeutic agents.

Natural compounds present as potential antifungal agents, as they are readily available in many foods and plants sources, and have been known in traditional medicine for their antimicrobial, anti-inflammatory, and antioxidant effects (Martins et al., 2009). Monolaurin is a monoglyceride, composed of lauric acid esterfied with glycerol, and is found in coconut oil (Carpo, Verallo-Rowell & Kabara, 2007). It is important to note that monolaurin is recognized as GRAS (Generally Recognized as Safe), as a food additive by the FDA (Food and Drug Administration), with topical doses of up to 100 mg/ml (Title 21, Code of Federal Regulations, Part 184) (Peterson & Schlievert, 2006). Studies have shown that monolaurin has broad bioactivities, such as antibacterial and antiviral properties (Bergsson et al., 2001; Carpo, Verallo-Rowell & Kabara, 2007; Li et al., 2009; Peterson & Schlievert, 2006). In an *in vitro* study by Schlievert & Peterson (2012), the antibacterial activity of glycerol monolaurate (GML) was tested against gram-positive bacteria, *Staphylococcus aureus*. GML prevented biofilm formation of *S. aureus* with no drug resistance developing at a sub-growth inhibitory concentration (Schlievert & Peterson, 2012). It was concluded that GML has a broad-spectrum of antimicrobial activity and has the potential for future application as a topical therapeutic agent *in vivo*. However, to the best of our knowledge, the antifungal activity of monolaurin against *Candida albicans* biofilms has never been studied.

For the first time, this study aims to primarily evaluate the antifungal activity of monolaurin against *Candida albicans* biofilms *in vitro* and to investigate whether monolaurin can modulate host immune response during fungal infections. Furthermore, we provide validation for the safety of monolaurin use in future *in vivo* study for the treatment of oral candidiasis by assessing the cytotoxicity of monolaurin on oral fibroblast cells. Finally, given the fact that proteolytic enzymes secreted by *C. albicans* are often reported to elicit host tissue damage and are thus considered key virulence factors in the pathogenicity of *C. albicans*, we examined whether monolaurin had any modulatory effects on the enzymatic activities levels of proteinases and phospholipases enzymes.

## Materials & Methods

### Susceptibility test

The antifungal activity of 1-monolaurin (Sigma) was tested *in vitro* against the following *Candida albicans* strains: fluconazole-resistant strain 96901, SC5314, ATCC: MYA2876, and ATCC 90028, according to the NCCLS M27-A protocol (National Committee for Clinical Laboratory Standards (NCCLS), 2002; Pasetto, Pardi & Murata, 2014). The strains of *C. albicans* are proven virulent pathogens and were selected for their known genomic sequencing (Jones et al., 2004). The minimum inhibitory concentration (MIC) was determined using planktonic *C. albicans* inoculum of 5 × 10^3^ CFU/ml, which was confirmed using a spectrophotometer by measuring an absorbance in the range of 0.08–0.10 at 625 nm. *C. albicans* inoculum was grown in RPMI-1640 (Lonza) in a 96-well plate. Serial dilutions in the range of 3.9–2,000 µM of 1-monolaurin (99.9% high-performance liquid chromatography purchased from Sigma) were prepared. Based on the MIC found for the tested *C. albicans* strains, fluconazole (32.2 µM) (Sigma) was used as the positive control in this experiment in comparison to the vehicle control 1% Ethanol (v/v). The plate was incubated for 24 h at 37°C in 5% CO_2_. Minimum inhibitory concentration (MIC) was determined after 24 h as the concentration, at which *C. albicans* growth was visibly inhibited (National Committee for Clinical Laboratory Standards (NCCLS), 2002; Pasetto, Pardi & Murata, 2014). Minimum fungicidal concentration (MFC) was found by subculturing 20 µl of each well on Sabouraud Dextrose Agar (BD). After 24 h of incubation, MFC concentration was determined as the lowest concentration of 1-monolaurin, showing no visible *C. albicans* growth on the agar plates (National Committee for Clinical Laboratory Standards (NCCLS), 2002; Pasetto, Pardi & Murata, 2014).

### Biofilm assay

An inoculum of 1 × 10^6^ CFU/ml of *C. albicans* (ATCC: SC5314/MYA2876) was grown for 24 h in a sterile 24-well plate using Yeast Nitrogen Base Medium (Difco) with 50 mM of glucose for 24 h at 37°C in 5% CO_2_ to establish initial biofilm growth. Total volume of 1 ml of inoculum was pipetted in each well. After 24 h of incubation, the biofilms were treated once daily with 100 µl of 1-monolaurin at concentrations of 1,250 µM and 2,500 µM (equivalent to 10×MIC and 20×MIC; respectively), which remained incubated with the biofilms suspended in medium overnight. The vehicle control used was 1% ethanol while the positive control was fluconazole (322 µM; equivalent to ten times the MIC). Before each treatment, biofilms were washed with Phosphate Buffer Solution (PBS) and replenished with 900 µl of fresh medium in addition to 100 µl of the corresponding treatment, yielding a total volume of 1 ml in each well. After 72 h of treatments, biofilms were suspended in PBS and the solution was centrifuged at 10,000 rpm for 5 min (Santana et al., 2013). Colony formation unit (CFU) was determined by suspending each sample of biofilm in 1 ml of PBS and plating 20 µl of the suspension on Sabouraud Dextrose Agar plates (BD). After 24 h of incubation, the number of *C. albicans* colonies was counted and data was expressed in Log (CFU/ml).

### Proteinase and phospholipase enzyme secretion assay

Proteinase and phospholipase enzyme secretion assays were conducted as previously performed by Santana et al. (2013). Biofilms of *C. albicans* were grown for 24 h in Yeast Nitrogen Base Medium (Difco) with 50 mM of glucose at 37°C in 5% CO_2_ and treated using 1-monolaurin concentrations of 1,250 µM and 2,500 µM (10× MIC and 20× MIC; respectively). Phospholipase A2 (for phospholipase assay) (Sigma) and Trypsin (for proteinase assay) (Lonza) were used as standards. The vehicle control used in this assay was 1% ethanol. After 72 h of biofilm maturation, the enzyme secretion assays were performed on biofilms suspended in PBS, which were sonicated for 15 s at 20% amplitude with pulses at 5 s and 10 s intervals (FB120; Fischer Scientific, Pittsburgh, PA, USA). The proteinase enzyme activity was determined by mixing the supernatant of the biofilm solution with 1% azocasein at 1:9 (v/v) for 1 h at 37°C in 5% CO_2_. Then, 500 µl of 10% trichloroacetic acid was added to stop the reaction. The solution was centrifuged for 5 min at 10,000 rpm and 500 µl of the supernatant was combined with 500 µl of NaOH, which was incubated for 15 min at 37°C in 5% CO_2_. Absorbance was read in a spectrophotometer at 440 nm (Goncalves et al., 2012; Pande et al., 2006; Santana et al., 2013). The phospholipase enzyme activity was determined by mixing the supernatant of the biofilm solution with phosphatidylcholine substrate for 1 h at 37 °C in 5% CO_2_ and reading the absorbance in a spectrophotometer at 630 nm (Goncalves et al., 2012; Santana et al., 2013; Taniguchi et al., 2009).

### Co-culture model fluorescence microscopy

Cytotoxicity assays using fluorometric quantification of cellular viability were performed on oral fibroblast cells (ATCC: CRL2014). Fibroblast cells (1 × 10^5^ cells/ml) were first seeded in a 96-well plate in DMEM medium with 10% FBS and incubated at 37°C in 5% CO_2_ for 24 h. Cells were then treated with 1-monolaurin (3.9–2000 µM) and incubated for an additional 24 h. Cell viability and morphological characteristics were observed by fluorometric method and microscope, respectively (National Committee for Clinical Laboratory Standards (NCCLS), 2002; O’Brien et al., 2000; Pasetto, Pardi & Murata, 2014). The medium was then replaced with an inoculum of 5 × 10^3^ to 2.5 × 10^3^ CFU/ml *C. albicans* (ATCC: SC5314) grown in DMEM without FBS. Fibroblast cells and *C. albicans* were treated with 125 µM and 250 µM of 1-monolaurin. The plate was then incubated for 24 h. The vehicle control tested was 1% ethanol while the positive control used was fluconazole (32.2 µM). The distribution of dead and live fibroblast cells was examined using the Viability/Cytotoxicity Assay Kit for Animal Live & Dead Cells (Biotium), which contains a mixture of Calcein AM and EthDIII. Calcofluor white (Sigma) was used to stain *C. albicans*. Fluorescent images of the double staining were captured using fluorescence microscopy (EVOS fl microscope AMG, Bothell, WA, USA).

### Co-culture model quantitative real-time PCR

All RNA was isolated from fibroblast cells and *C. albicans*. The fibroblast cell RNA and *C. albicans* RNA were isolated and purified using the RNeasy MiniKit (Qiagen) and the Ribopure Yeast Kit (Life Technology); respectively. A NanoPhotometer P360 (Implen) was used to quantify the total RNA extracted. Reverse transcription of the RNA into cDNA was carried out using iScript Advanced cDNA synthesis Kit for RT-qPCR (Biorad). Real-time PCR was conducted by using iQ SYBR Green Supermix (Biorad). The *C. albicans* primers for the genes: *Secreted Aspartyl Proteinases-1 (SAP-1), Phospholipase B-1 (PLB-1),* and *ACT-1 (housekeeping)* were used (Nailis et al., 2010). *ACT-1* was the gene used to normalize *SAP-1* and *PLB-1* genes expression. Based on a previous analysis using the RT2 Profiler PCR Array Kit (Qiagen), the following host inflammatory cytokines genes were selected: IL1-alpha (Qiagen Gene ID#: 3552; Qiagen, Hilden, Germany), IL1-beta (Qiagen Gene ID#: 3553; Qiagen, Hilden, Germany), IL-8 (Qiagen Gene ID#: 3576; Qiagen, Hilden, Germany) and *GADPH* (housekeeping) (Qiagen Gene ID#: 2597; Qiagen, Hilden, Germany). All data from cytokines genes expression were normalized using the housekeeping gene *GADPH*. PCR amplification was performed by using 20 µl reaction mix per well in a 96-well plate. The reactions were conducted at 95°C for 3 min, followed by 40 cycles of 15 s at 95°C and 1 min at 60°C. After PCR, the melting curve was obtained by incubating the samples at increasing increments of 0.5°C from 55°C to 95°C.

### Statistical analysis

All data were expressed as means ± SEM using One-way Analysis of Variance (ANOVA), followed by Dunnett test. The level of statistical significance was set at 0.05. Results were considered significant if p-values were less than 0.05.

## Results

Susceptibility assay of 1-monolaurin showed antifungal activity against several strains of *C. albicans* in planktonic form, including a fluconazole-resistant strain 96901 in comparison to a standard antifungal agent, fluconazole (Table 1). The ranges of the minimum inhibitory concentrations (MIC) and the minimum fungicidal concentrations (MFC) of monolaurin against *C. albicans* MYA 8276 were compared to those of fluconazole. The MIC and MFC of monolaurin were in the ranges of 62.5–125 µM and 125–250 µM, respectively, while the MIC and MFC of fluconazole; were 32.2 µM and 100 µM, respectively. The MIC and MFC of monolaurin against the fluconazole-resistant strain 96901 were found to be much lower than those of fluconazole; 30 µM and 140 µM in comparison to the MIC and MFC values of fluconazole of 100 µM and 350 µM, suggesting a strong antifungal potential against fluconazole resistant strains. The MIC and MFC were established to identify the concentrations needed to be applied to biofilms, which were in the range of ten times the MIC.

**Figure 1:**
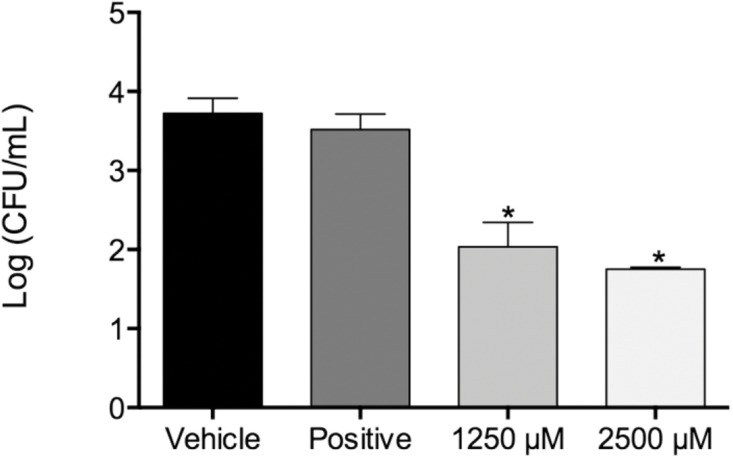
Fungal load of biofilms treated with monolaurin and proteolytic enzymes activity level. Fungal load of 1-monolaurin treated biofilms. *p < 0.05.

**Table 1:** Minimum inhibitory concentration (MIC) and minimum fungicidal concentration (MFC) of 1-monolaurin against *Candida albicans*.

Microorganism	Monolaurin		Fluconazole (positive control)	
	MIC (µM)	MFC (µM)	MIC (µM)	MFC (µM)
*Candida albicans* 96901^a^	30	140	100	350
*Candida albicans* SC5314	25	100	10	80
*Candida albicans* MYA-2876	62.5–125	125–250	32.2	100
*Candida albicans* 90028	20	110	20	90

**Notes.**

a Fluconazole resistant.

In the biofilm assay, monolaurin in the concentrations of 1,250 µM and 2,500 µM, equivalent to ten times and twenty times the upper range of MIC, were tested on *C. albicans* MYA 8276. Biofilm fungal load was expressed, as the Log of the colony formation unit (CFU/ml). The results showed that treatments with 1-monolaurin at 1,250 µM and 2,500 µM had significant reduction (p < 0.05) in fungal load in comparison to the vehicle control group (Fig. 1). Such findings indicate a strong potential antifungal activity of monolaurin against *C. albicans* biofilms. It is important to note that 1-monolaurin (3.9–2,500 µM) presented very minimal cytotoxicity to oral fibroblast cells, as illustrated in Fig. 3. At the highest concentration of 2000 µM shown in Fig. 3, approximately 50% of fibroblast cells were viable. Thus, the findings of the cytotoxicity assay indicated that monolaurin in the concentrations of 3.9–2,500 µM did not result in 0% viability of cells. Such findings confirmed the safety of monolaurin to oral cells, especially as it has been approved by FDA as “GRAS” food additive substance.

**Figure 2:**
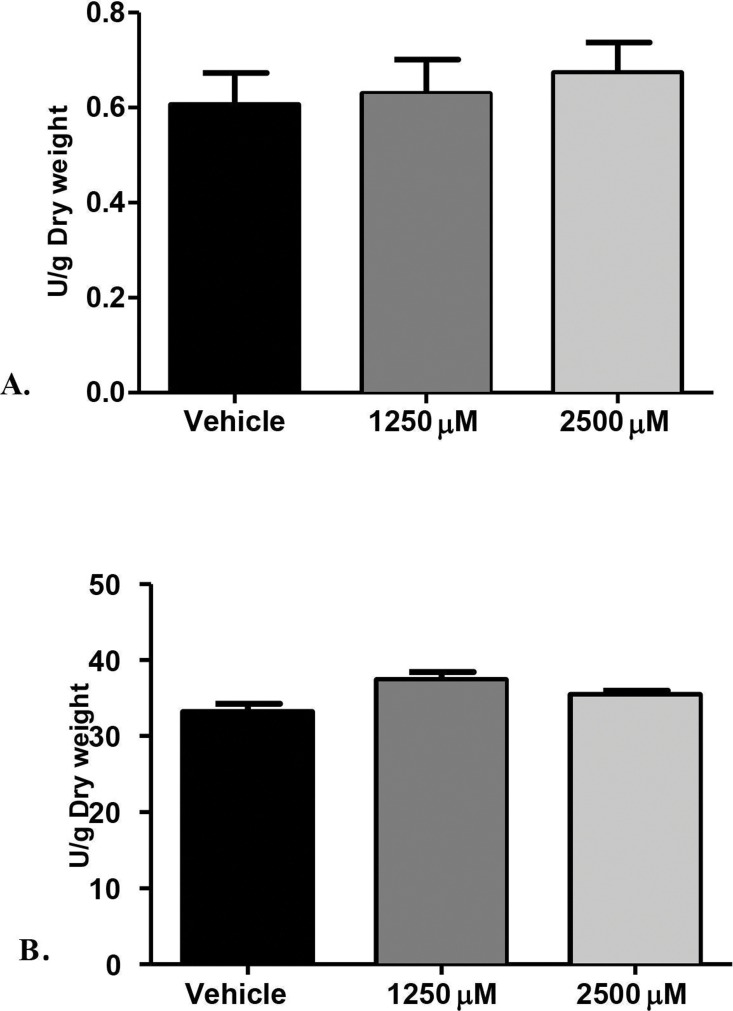
Proteolytic enzymes activity of *C. albicans* after treatment with monolaurin. Secreted proteolytic enzymes activity of *Candida albicans* after treatment with 1-monolaurin; of (A) Proteinase, and (B) Phospholipase enzymes.

Furthermore, we examined the enzymatic activities of *C. albicans* secreted proteolytic enzymes, in the proteinase and phospholipase enzyme assays of biofilms, as such enzymes are often associated with host tissue damage. Our goal was to identify any modulatory effects of monolaurin on the enzyme activity level of *C. albicans* biofilms, which are considered a major virulence factor contributing to the pathogenicity of *C. albicans* biofilms. Collected supernatants of biofilms, which were treated with 1,250 µM and 2,500 µM of 1-monolaurin, showed no significant difference in either enzyme activity of proteinases or phospholipases, when compared to the vehicle group (Fig. 2). Thus, there was no reduction in enzyme activities, as expressed in Unit U/g of biofilm dry weight. Such findings suggest no modulatory effects of monolaurin on either the proteinase or the phospholipase enzymes.

In addition, co-culture models of immature biofilms of *Candida* coexisting with fibroblasts exposed to monolaurin treatments of 62.5 µM and 125 µM were useful in assessing the gene expression of *Secreted Aspartyl Proteinases-1 (SAP-1)* and *Phospholipase B-1 (PLB-1)*, two common genes that are often up-regulated in the presence of elevated enzymatic activities of proteinases and phospholipases. Total RNA isolated from *Candida* in the co-culture model was reverse transcribed into cDNA, which was used in real-time PCR to quantify the gene expression of *SAP-1* and *PLB-1* normalized to a house keeping gene, *ACT-1*. Similar to the results obtained from the biofilm proteinase and phospholipase assays, there was no significant down-regulation of either *SAP-1* or *PLB-1* in the groups treated with 62.5 µM and 125 µM of monolaurin compared to the vehicle control (Figs. 5A and 5B). Such findings confirm that there was no gene up-regulation of either *SAP-1* or *PLB-1* in the immature *C. albicans* treated with monolaurin (62.5 µM and 125 µM).

**Figure 3:**
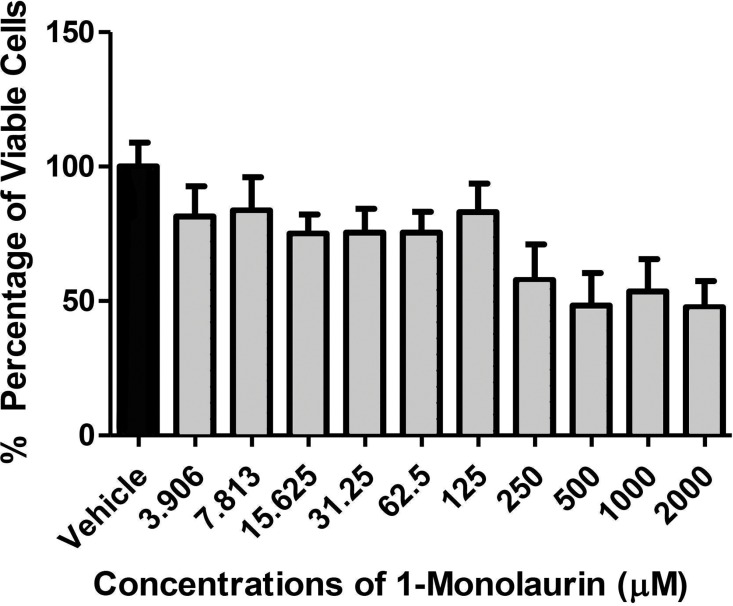
Cytotoxic effects of 1-monolaurinon on oral fibroblast cells. Note that monolaurin is recognized as GRAS (Generally Recognized as Safe), as a food additive by the FDA (Food and Drug Administration), with topical doses of up to 100 mg/ml (Title 21, Code of Federal Regulations, Part 184).

**Figure 4:**
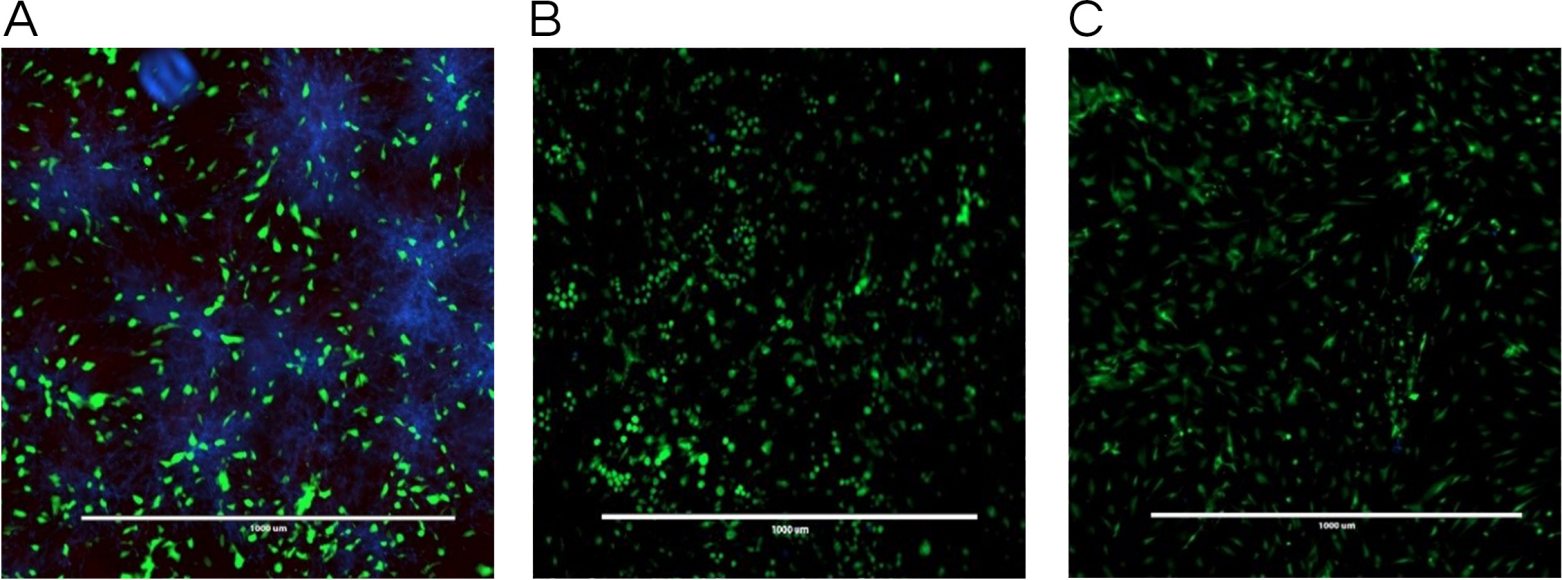
Co-culture fluorescence microscopy of 1-monolaurin. (A) vehicle control (1% ethanol), (B) positive control (fluconazole), and (C) 1-monolaurin (125 µM); stained with calcofluor white and cytotoxicity assay kit for animal Live/Dead cells (Blue: *Candida albicans*, Green: live fibroblast cells, and Red: dead fibroblast cells). Scale bar set at 1,000 um at 4×magnification power.

The co-culture model was also helpful in visualizing a real model of immature *C. albicans* biofilm coexisting with fibroblast cells incubated with a tested antifungal treatment. Images obtained by fluorescence microscopy allowed for a qualitative assessment of the distribution of *C. albicans* (blue color) with respect to live (green color) and dead (red color) fibroblast cells. Figure 4 showed fluorescent imaging of *C. albicans* coexisting with oral fibroblasts in the presence of treatment of monolaurin (125 µM) in comparison fibroblasts and *C. albicans* treated with vehicle control (1%) and positive control (fluconazole). Co-culture model treated with 1-monolaurin (125 µM) (Fig. 4C) showed sparse and less dense accumulation of *C. albicans* (blue) compared to that treated with a vehicle control, 1% ethanol (Fig. 4A). It should be noted there were some dead fibroblast cells present in the monolaurin treated models (Fig. 4C) since the cells in this model were considered more sensitive to the agent being exposed to as opposed to cells examined in complex clinical systems. Such observation of some dead cells detected in the co-culture model of monolaurin are in agreement with the cytotoxicity reported for monolaurin, where there was approximately 80% cell viability found at 125 µM of monolaurin. In general, co-culture images of less dispersed *C. albicans* in the presence of monolaurin (Fig. 4C) were comparable to those of the positive control group (fluconazole 32.2 µM) in Fig. 4B, suggesting the efficacy of monolaurin in inhibiting the growth of *C. albicans* biofilms.

**Figure 5:**
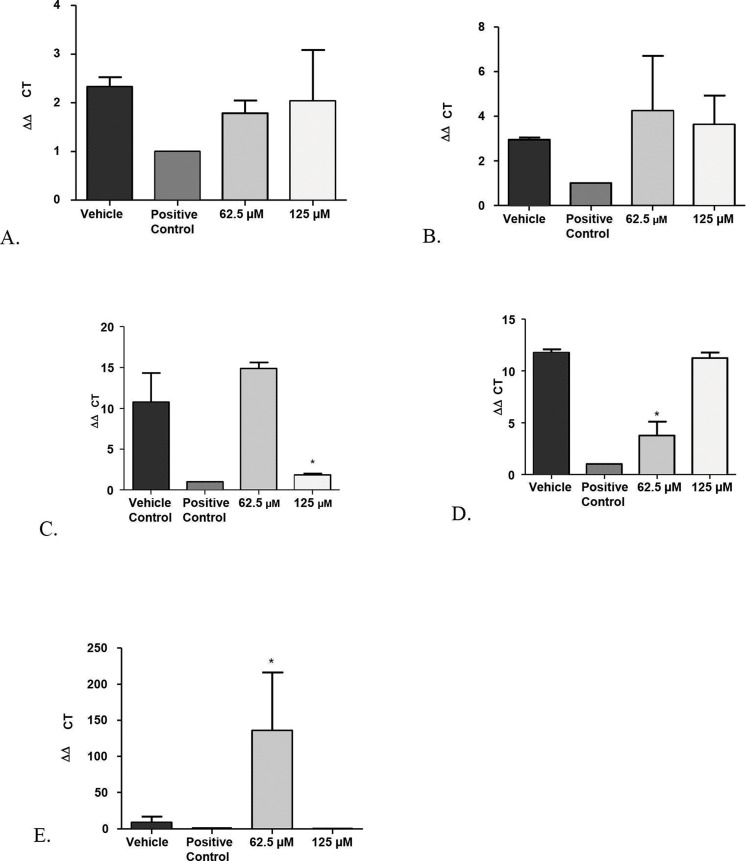
Real-time quantitative gene expression of oral fibroblast cells infected by *C. albicans* after 1-monolaurin treatments at 62.5 µM and 125 µM concentrations in comparison to vehicle control and positive control fluconazole (32.2 µM). (A) *SAP-1* (B) *PLB-1* (C) IL-1*α* (D) IL-1*β* (E) IL-8.

Another advantage of the co-culture model was that it provided invaluable information on gene expression of host fibroblast cells infected with *C. albicans* and exposed to 1-monolaurin treatments. Quantitative Real-time PCR assessed host gene expression of pro-inflammatory cytokines IL-1alpha, IL-1beta, and IL-8 in samples treated with of 1-monolaurin at MIC concentrations of 62.5 µM and 125 µM in comparison to the vehicle control and the positive control groups. The goal of examining the cytokines expression of the host was to test if there was any modulatory effects of monolaurin on the host’s pro-inflammatory response during fungal infection. Figure 5C showed that at 125 µM of monolaurin, there was significant down-regulation of IL-1*α* compared to the vehicle control group (p < 0.05). However, at a lower concentration of 62.5 µM, there was no down-regulation of the IL-1*α* gene expression. Figure 5D showed that at 62.5 µM, there was a significant down-regulation of IL-1*β* gene expression in comparison to the vehicle control group. However, there was no similar down-regulatory effect at a higher concentration of 125 µM. The down-regulation of the pro-inflammatory cytokines gene expression of IL-1*α* and IL-1*β* found with the treatments of monolaurin suggested that monolaurin was capable of modulating the host inflammatory response although not in a dose dependent way, as in the case of IL-1*β* gene expression. IL-8 is another pro-inflammatory cytokine known as a neutrophil chemotactic factor that induces migration of neutrophils and phagocytosis at the infection site (Villar et al., 2005). We hypothesized that monolaurin may affect the pro-inflammatory IL-8 cytokine in a down-regulatory manner similar to the pro-inflammatory cytokines IL-1*α* and IL-1*β*. However, Fig. 5E showed a significant up-regulation of IL-8 in the group treated with 62.5 µM of 1-monolaurin compared to the vehicle control group, suggesting a strong neutrophilic chemotactic activity by the host cells. These results were not dose dependent since at higher concentration of monolaurin (125 µM), there was a down-regulation of IL-8. However, the results of the down-regulation of IL-8 by monolaurin at 125 µM were not statistically significant when compared to the vehicle control group.

A possible explanation for the up-regulation of IL-8 noted in the samples treated with 62.5 µM of 1-monolaurin may be due to a negative feedback inhibition resulting from the down-regulation of IL-1, as it was found that IL-1 induces IL-8 secretion in fibroblasts (Kaplanski et al., 1994). Thus, by the decrease in the production of IL-1 as expected by the down-regulation of IL-1 gene expression, there may be a negative feedback inhibition of IL-8, resulting in the up-regulation of the gene expression of IL-8. It should be noted that these cytokines were analyzed at an early or acute phase of fungal infection. It would be interesting to assess such cytokines gene expression in a future model simulating a more chronic phase of infection.

## Discussion

The primary goal of this study was to evaluate the antifungal effect of monolaurin against *C. albicans* biofilms *in vitro* using a co-culture model. In addition, we investigated whether monolaurin can alter the morphology of the biofilm community as well as the proteolytic enzymatic activities of proteinases and phospholipases, which are considered critical virulence factors associated with the pathogenicity of *C. albicans.* Furthermore, we explored the modulatory effects of monolaurin on the pro-inflammatory cytokines gene expression of host fibroblast cells.

Susceptibility tests of monolaurin against *C*. *albicans* (MYA2876) showed inhibition of fungal growth at an MIC range of 62.5–125 µM while MFC was in the range of 125–250 µM, which showed potential antifungal activity against this specific tested strain. In the biofilm assay model, higher concentrations of monolaurin in the range of 10× and 20× the concentrations of MIC, were used due to the tenacious nature of biofilms to be eradicated. Biofilms treated with monolaurin showed significant reduction (p < 0.05) in the fungal load, as illustrated by the decrease in Log (CFU/ml) in comparison to the control groups.

Similarly, the results obtained by fluorescence microscopy of the co-culture model (Fig. 4C) showed a large decrease in viable *C. albicans* dispersed among oral fibroblasts in the presence of 1-monolaurin (125 µM). It is important to note there were some morphological changes in the oral fibroblast cells of the positive control group treated with fluconazole (Fig. 4B) as well as those of the monolaurin treated group (Fig. 4C) in comparison to the cells of the vehicle control group. While the overall density and the distribution of live fibroblasts (green color) may seem to be similar to the vehicle control, there may be a shortened or a less elongated appearance of the fibroblasts in the 2 tested groups, of monolaurin and fluconazole. A possible explanation is that a co-culture model of fibroblasts coexisting with *Candida* and an antifungal agent may present an exaggerated, sensitive effect on the morphology and distribution of fibroblast cells compared to cells tested under clinically relevant conditions. Thus, the oral fibroblast cells in co-culture model are considered to be “naked cells,” with more sensitivity to the treatments administered. Therefore, the cells may show more signs of stress when exposed to an antifungal agent compared to those of the vehicle control group.

As mentioned earlier, the safety of monolaurin, as a food additive and emulsifier was recognized as GRAS (Generally Recognized as Safe) by the FDA (Food and Drug Administration) for up to 100 mg/ml (Peterson & Schlievert, 2006). Not surprisingly, in our cytotoxicity assay, 1-monolaurin showed no toxicity to oral fibroblast cells. In this study, the lethal dosage of 1-monolaurin could not be calculated from a regression analysis since no concentration of monolaurin has resulted in 0% cell viability. Thus, 1-monolaurin showed no toxicity (up to 2,500 µM) to oral fibroblast cells and therefore was considered safe to be studied in future *in vivo* investigations.

A major helpful analysis provided by the co-culture model was to study the inflammatory cytokines of the host by quantification of a panel of inflammatory markers, such as IL-1*β*, IL-6, IL-8, IL-10, and IL-17 (Arien, Vanham & Gali, 2011). Monolaurin 5% gel was previously shown to inhibit innate inflammatory responses, as it prevented vaginal SIV transmission in monkeys (Li et al., 2009). More specifically, glycerol monolaurate has inhibitory activity against the production of MIP-3*α* and other pro-inflammatory cytokines, and can inhibit mucosal signaling and block the inflammatory response to HIV-1 and SIV *in vitro* and *in vivo* (Li et al., 2009). In our current study, we tested gene expression of pro-inflammatory cytokines, IL-1*α* and IL-1*β* after treatments with 1-monolaurin at 62.5 µM and 125 µM. Our results showed a significant (p < 0.05) down-regulation of IL-1*α* with the treatments of 1-monolaurin treatments at 125 µM (Fig. 5C), while down-regulation of the IL-1*β* gene expression was achieved with monolaurin treatments at 62.5 µM. However, gene expression of IL-8 was not down-regulated in the biofilms treated with monolaurin. It can be concluded that monolaurin can modulate host response by down-regulating the gene expression of some of the pro-inflammatory cytokines, such as IL-1*α* and IL-1*β*. Future research interests may involve studying other pro-inflammatory as well as anti-inflammatory markers, such as IL-10, using the same co-culture model in order to provide a more comprehensive analysis of the drug’s ability to modulate host response and to determine if it can contribute to the anti-inflammatory mechanism in mammalian cells.

The rationale for studying the activity of proteinases and phospholipases was that such hydrolytic enzymes have been reported in the literature to be secreted by *C. albicans* and are known to elicit host tissue damage (Lermann & Morschhauser, 2008). Thus, these proteolytic enzymes are considered key virulence factors in the pathogenicity of *C. albicans.* Our hypothesis was that if monolaurin were to have an antifungal activity against *Candida*, it might do so by down-regulating such proteolytic enzymes. However, our findings did not confirm such hypothesis. It was found that treatments of monolaurin at 1,250 µM and 2,500 µM were not associated with a decrease in the enzymatic activity level of either the proteinases or the phospholipases (Figs. 2A and 2B).

To confirm the findings on the proteolytic enzyme activities, the gene expression of *SAP1* (Secreted aspartyl protease) and phospholipase B (*PLB*) were assessed. During fungal infections, there is generally a higher gene expression of *SAPs* (1-10), which is often associated with hyphal formation and induction of rim101p, a transcription factor that mediates the degradation of E-cadherin protein of the epithelial cell junction (Naglik et al., 2008). In our study, the gene expression of *SAP-1* was evaluated after application of treatments of 1-monolaurin at concentrations of 62.5 µM and 125 µM and normalized by the expression of *ACT*, a housekeeping gene. There was no statistical significance difference in *SAP-1* gene expression in comparison to the control groups (Fig. 5A). However, the role of specific *SAP* genes to their attenuated phenotype is yet to be elucidated. In addition, more studies would be helpful to explore the effects of monolaurin on the other members of the SAPs family.

Similarly, phospholipases B1, B2, C and D of *C. albicans* play a significant role in the invasion of the host tissue, as noted by their high gene expression during fungal infection (Samaranayake et al., 2006). More specifically, phospholipase B (PLB) proteins were shown to have hydrolytic activity, as they hydrolyze acyl ester bonds in phospholipids and lysophospholipids and catalyze lysophospholipase-transacylase reactions (Theiss et al., 2006). It was determined that the *PLB* multigene family of the opportunistic fungal pathogen *C*. a*lbicans* encodes for CaPLB5, a putative secretory protein with a predicted GPI-anchor attachment site. The ability of *C. albicans* to attach itself to the host tissue is considered a key pathogenic characteristic and hence, genes encoding for attachment proteins, such as *PLB*, may be potential virulence determinants (Theiss et al., 2006). In this study, phospholipase enzyme activity was tested using both 1,250 µM and 2,500 µM concentrations of 1-monolaurin, which showed no significant difference when compared to the vehicle group (Fig. 2B). More studies are needed to elucidate the role of monolaurin on the gene expression of the other phospholipases, such as phopspholipases B2, C, and D. Thus, it can be concluded that monolaurin did not have a down-regulatory effect on the gene expression of either *SAP-1* or *PLB-1* encoding for their respective enzymes produced by *C*. *albicans.*

In conclusion, 1-monolaurin had potential antifungal activities against *Candida albicans* both in susceptibility tests and biofilm assays. Furthermore, monolaurin had immune-modulatory effects on the host cells, as indicated by its down-regulation of pro-inflammatory cytokines gene expression of IL-1*α* and IL-1*β*. Future direction for research may include understanding its impact on proteases activity on the cellular level and whether there is a direct effect on attachment protein gene expression. Ultimately, future studies may validate the efficacy of monolaurin *in vivo*, which may translate into its potential clinical use to prevent and/or treat oral candidiasis.

##  Supplemental Information

10.7717/peerj.2148/supp-1 Supplemental Information 1 Biofilms fungal load results with monolaurin treatments

10.7717/peerj.2148/supp-2 Supplemental Information 2 Real-time PCR Quantitative analysis of virulence factors of *C. albicans* and fibroblasts inflammatory cytokines gene expression after treatment with monolaurin

10.7717/peerj.2148/supp-3 Supplemental Information 3 Toxicity of monolaurin on fibroblasts

10.7717/peerj.2148/supp-4 Supplemental Information 4 Minimum inhibitory concentration (MIC) of monolaurin on *C. albicans*

10.7717/peerj.2148/supp-5 Supplemental Information 5 Proteolytic enzyme analysis of *C. albicans* proteinase and phospholipase after biofilms treatments with monolaurin

## Additional Information and Declarations

### Data Availability

The following information was supplied regarding data availability: The raw data has been supplied as Supplemental Information.
